# Society Meetings

**Published:** 1917-01

**Authors:** 


### SOCIETY MEETINGS

Brief reports and announcements of meetings of societies of Western New York. and those of general scope, are requested from Secretaries. Copy should be on hand the fifteenth of the month. Full transactions will be published at cost of composition.

**DOCTOR: Is your Society properly represented here? If not, please bring our standing announcement to the attention of the members.**

**The Chautauqua County Medical Society** met at Dunkirk Dec. 13. Officers were elected as follows: Pres., J. H. Kellogg, Bemus Point; V. P., Walter Stewart, Westfield and N. E. Beardsley, Dunkirk; Sec., J. W. Morris, Jamestown; Treas., G. F. Smith, Falconer; Censor and Delegate to State Society, V. M. Griswold, Fredonia; Alternate, A. W. Dods, Fredonia.

**The Elmira Academy of Medicine** held its annual meeting Dec. 2. Dr. LaRue Colgrove gave as his presidential address a paper on Acidosis, which will be published in this journal later, and Dr. F. L. Christian gave a paper on Germ Plasm and Heredity. Officers were elected as follows: Pres., Reeve B. Howland; V. P., Elliott T. Bush; Sec., O. J. Bowman; Treas., Abraham Lande; Trustees, A. H. Baker and G. V. R. Merrill. Dr. R. O. Baker of Montour Falls and Dr. Floyd Breese of Elmira were elected to membership.

**The Rochester Academy of Medicine,** Section iv., met Dec. 13. The program was as follows: a. "The Relation of Physicians to Health Insurance.'' Dr. Albert Bowen; discussion opened by Dr. William B. Jones, Dr. Edward G. Whipple.

b. ''The Consideration of the Workmen's Compensation Laws from the Standpoint of the Practitioner of Medicine and Surgery,'' Dr. Harold Baker; discussion opened by Dr. William H. Cadmus, Dr. Guy L. Howe, Dr. George M. Gelser.

Myron B. Palmer, M. D., Chairman; Curtiss N. Jameson, M. D., Secretary.

---

**Buffalo Academy of Medicine.** Stated meeting Nov. 29, with Surgical Section: By-laws, Art. 1 was amended to read: Section 2. ''At the stated meeting in March each year for the nomination of officers of the Academy, the date of the annual meeting shall be determined and such date shall not be earlier than June 1, the ending of the fiscal year, and not later than June 15. Program: Exhibition of bone graft cases by Dr. Prescott LeBreton; Symposium on Head Injuries: End results with and without fracture, Dr. James W. Putnam; Treatment with and without fracture, Dr. Herbert A. Smith; Diagnosis with and without fracture, Dr. Marshall Clinton;.

Special meeting Dec. 6 to consider and interpret the portion of Section 1, Article 6, of the constitution ''for unprofessional or dishonorable conduct,'' special reference being had to fee splitting. A regular meeting of the Medical Section followed, Dr. Chas. G. Stockton giving a lantern slide demonstration of Unusual Stomach Conditions.

Section of Obstetrics and Gynaecology, Dec. 13: Indications and Contra-indications for Caesarian Section, Wm. T. Getman; Plea for the Immediate Repair of Obstetric Injuries, Ray H. Johnson, discussion being led by Drs. Potter and Van Peyma.

---

**The Gross Medical Club** held its stated meeting at the Hotel Markeen, being entertained by Chester C. Cott. Dr. Charles McKay read a paper ''The high caloric feeding in typhoid,'' (will be published later). Several very interesting and important cases were reported by Drs. Bennett, G. F. Cott, G. A. Phelps (E. Aurora) and others. After dinner, which closed the sessions of 1916, the members and their wives reassembled at the Teck Theater where Drs. Cott and Dowd provided seats for ''Just a Woman.''

---

**American Medical Editors' Association.** The officers elected for 1916-17 were as follows: President, Geo. M. Piersol, M. D., Phila., Pa. (Editor Amer. Jour. Med. Sciences); 1st V. P., Geo. W. Kosmak, M. D., New York (Editor Amer. Jour. of Obstetrics); 2d V. P., Robert M. Green, M. D., Boston, Mass. (Editor Boston Med. and Surg. Jour.); Sec. and Treas.,

J. McDonald, Jr., M. D., N. Y. (Mgr. Editor Am. Jour. of Surg.); Ex. Com., C. F. Taylor, M. D., Phila. Pa. (Editor Medical World); A. S. Burdick, M. D., Chicago, Ill. (Editor Am. Jour. Clin. Med.); D. S. Fairchild, M. D., Clinton, Iowa (Jour. Iowa State Med. Society). Dr. H. Edwin Lewis, N. Y., (Editor Am. Med.), chairman of the Publication Committee and Editor of the Jour. of the Am. Med. Editors' Association. The next annual meeting will be held in N. Y. with headquarters at the McAlpin Hotel on June 4th and 5th, 1917.

---

**The American Congress of Internal Medicine** announce its first scientific session at the Hotel Astor, N. Y. City, Dec. 28 and 29. The President, Reynold Webb Wilcox will give an address on the Domain of Internal Medicine and the Purport of the Congress. Symposiums on the Ductless Glands in Cradio-vascular Diseases and Dementia Precox and Duodenal Ulcer constitute the remainder of the program. In an accompanying circular letter, the following explanations of the purpose of the Congress are made:

Although the range and scope of Internal Medicine are well defined, it is a fact that the average practitioner of Medicine and Surgery, and the laity, do not differentiate between the General Practitioner and the Internist.

The trend of modern medicine is by no means toward specialism. A practitioner may devote especial attention to a certain limited field, but the days of the out-and-out stomach specialist, neurologist, pediatrist, etc., are numbered. The public today demands a thoroughly educated physician, an Internist, and not a practitioner who has trained himself in the superficialities of a closely circumscribed field. Specialists of this class can only exist in a transitional period. We know that all the organs of the body are in some way linked together, and we know that the physiology is one coherent process of activity and compensation. The Internist understands this connection. To him belong the day and the future.

The Internist is in the first instance a diagnostician. It is he who dominates or, who ought to dominate, the field. He determines whether or not the case is to be operated upon or treated by other means. It is the Internist to whom has fallen the heritage of the earlier physicians. He is the master, the others are his handmaidens.

The American Congress on Internal Medicine has been called into life, (1) to procure official standing of the Internist, (2) to define the scope of Internal Medicine in the United States, (3) to link the Internists together, (4) to induce a feeling of solidarity amongst them, (5) to further the interests of Internists in general, (6) to advance the science of Biological Medicine in all its features, and (7) to do for the

Internist what has already been done for the co-ordinate practitioner, the surgeon.

The Congress intends to publish various magazines which will cover the entire field of Internal Medicine.

The Congress will meet once a year. It is the purport of the organization that its meetings be largely clinical. At the same time, the great questions of the day, especially those pertaining to Internal and Biological Medicine, will be fully ventilated. For this purpose a referee and co-referee will be appointed by the Committee on Scientific Work some months before the meetings, whose business it shall be to report on the questions submitted to them. General discussions upon these questions will be a special feature of the Congress.

For the purpose of stimulating investigation in the science and practice of medicine and of elevating the standard of medical education in this country, the enactment of just medical laws, of promoting the establishment of a National Board of Health, and to enlighten and direct public opinion on the great problems of health and medicine, the American College of Physicians has been chartered.

It is contemplated that, as far as possible, the officers and councillors of the Congress, shall be also the officers and councillors of the College.

The councillors of the Congress may upon application, nominate any fellow of that body to fellowship in the American College of Physicians, provided he has done meritorious work in some field of Internal Medicine, as specified in the charter and by-laws of that organization.

The American College of Physicians convenes once a year, at the close of the Congress, at which time candidates are elected to fellowships in the College.

President, Reynold Webb Wilcox, M. D., L.L. D., D. C. L., New York; Vice-President, Elias H. Bartley, B. S., M. D., Ph. G., Brooklyn; Secretary-General, Heinrich Stern, M. D., L. L. D., New York; Treasurer, Augustus Caille, M. D., New York.

---

**The Medical Society of the County of Erie** held its annual meeting in the Buffalo Medical College on Monday, December 18th, at 8.15 p. m. The following twelve applicants were elected to membership: Dr. Eucarpio Pisani, 257 Front Avenue, Buffalo; Dr. Grace Joslin Shaver, 1225 Clinton St., Buffalo; Dr. J. M. Park, Chaffee; Dr. George C. Barone, 497 Niagara St., Buffalo; Dr. Marvin Israel, Lancaster; Dr. Alice Bennett, 26 Allen St., Buffalo; Dr. Homer A. Trotter, 473 Virginia St., Buffalo; Dr. Joseph F. Trudnowski, Emergency Hospital, Buffalo; Dr. Frank A. Trippe, 298 Niagara St., Buffalo; Dr. John W. Burton, Moses Taylor Hospital, Lackawanna; Dr. Leo Edward Reimann, Buffalo General Hospital; Dr. Eugene J. Hanavan, 3 Roanoke Park, Buffalo.

The resignation of Doctor David E. Wheeler was accepted, he having moved to Frederickton, N. B.

The minutes of the last regular meeting and the minutes of the several Council meetings were approved.

This approval carried with it the adoption of the substitute motion to take the place of the motion introduced by Dr. Wende at the regular October meeting, relative to increasing the dues to the State Society $1.00 per annum, making it $4.00 instead of $3.00. The substitute motion is as follows:

Whereas, Two of the purposes of the Medical Society of the State of New York as defined in the constitution Article 1 are "to federate and to bring into one compact organization the medical profession of the State of New York," and "to guard and foster the material interests of its members," and

Whereas, These two purposes are best promoted by the protection offered by qualified liability insurance as conducted by the Society and known as the "Alleged Malpractice Defense," and

Whereas, The Counsel of the Society is inadequately remunerated in view of the marked increase in the demand for "Alleged Malpractice Defense," and the counsel should have an assistant under salary by the Society, and

Whereas, The annual income of the Society is amply sufficient to meet the increased demands for "Alleged Malpractice Defense," therefore in consideration of these whereases, be it

Resolved, That the Medical Society of the County of Erie condemns as unnecessary and impolitic any increase in the "annual per capita assessment on each County Society at a uniform per capita rate throughout the State," as defined in Article VIII of the Constitution, and that it directs its officers and delegates to oppose any attempt to secure any such increase; and

Resolved, That the Medical Society of the County of Erie unanimously promote and support any effort not inconsistent with these resolutions to increase the annual allotment of funds of the Medical Society of the State of New York for the use of the counsel of the Society in the prosecution of "Alleged Malpractice Defense," and that it directs its officers and delegates to so act as to secure such increase.

Annual reports were presented by Dr. J. D. Bonnar, chairman of the Board of Censors; Dr. Harvey R. Gaylord, chairman of the Committee on Legislation; Dr. Nelson G. Russell, chairman of the Committee on Public Health; Dr. John V.

Woodruff, chairman of the Committee on Economics; Dr. Clayton Green, Secretary of the Milk Commission, and Dr. Albert T. Lytle, Treasurer.

Dr. Gaylord advocated the appointment of a paid representative at Albany to look after legislation affecting the medical profession. On motion of Dr. Wende the Society decided to approve the resolution offered by Dr. Cruickshank, of Brooklyn at the last meeting of the State Society, relative to the State Committee on Legislation.

At Dr. Woodruff's request a motion was adopted in which the Committee on Economics was empowered to increase its membership from three to seven, and also to add an associate committee to assist it in its work.

Dr. George F. Cott, a member of the State Committee on Economics, presented an outline of the proposed legislation relative to Compulsory Health Insurance.

On motion of Dr. Woodruff the Society decided to invite Dr. S. Kopetsky, chairman of the State Committee on Economics, to address the Erie County Society on this subject of Compulsory Health Insurance at a special meeting to be held in January.

Dr. Albert T. Lytle also paid an official visit to the Society as President of the 8th District Branch.

The election of officers resulted as follows: President, Dr. Irving W. Potter; 1st Vice-President, Dr. George F. Cott; 2d Vice-President, Dr. James E. King; Secretary, Dr. Franklin C. Gram; Treasurer, Dr. Albert T. Lytle; Censors, Dr. J. D. Bonnar, Dr. Francis E. Fronczak; Dr. Arthur W. Bennett, Dr. A. D. Carpenter, Dr. Frank A. Valente; Chairman of the Committee on Legislation, Dr. Harvey R. Gaylord; Chairman of Committee on Public Health, Dr. Nelson G. Russell; Chairman of Committee on Membership, Dr. Wm. F. Jacobs; Chairman of Committee on Economics, Dr. John V. Woodruff; Delegates to the Medical Society of the State of New York, Dr. Grover W. Wende, Dr. Harry R. Trick, Dr. Arthur W. Hurd, Dr. Irving W. Potter, Dr. Charles G. Stockton.

Dr. Franklin C. Gram, Secretary.

